# The prevalence of different types of occlusal relationships based on the type of terminal plane in primary dentition: a study among 3- to 6-year old children in Tbilisi, Georgia

**DOI:** 10.34172/joddd.2022.004

**Published:** 2022-05-29

**Authors:** Elene Golovachova, Tinatin Mikadze, Manana Kalandadze

**Affiliations:** ^1^Department of Orthodontics, Faculty of Clinical and Translational Medicine, Tbilisi State University, Tbilisi, Georgia; ^2^Pediatric Dentistry Department, Faculty of Medicine, Tbilisi State University, Tbilisi, Georgia

**Keywords:** Primary dentition, Primary molars, Occlusion

## Abstract

**Background.** Primary dentition takes the most important role in the development of permanent dentition. Primary second molars determine the eruption and position of permanent first molars. Early diagnosis and treatment could prevent the establishment of occlusal anomalies and deformities, therefore this study aimed to collect data about the prevalence of malocclusions based on the deciduous second molar terminal planes among the Tbilisi preschoolers.

**Methods.** A total of 396 children aged 3-6 years, with an equal number of male and female subjects, were examined in kindergartens drawn from ten districts of Tbilisi, using the PPS (probability proportional to size) method. The relationship between distal surfaces of primary second molars was evaluated and recorded according to Baume’s classification. The significance level is 0.05 for all statistical tests.

**Results.** Out of investigated preschoolers, 52.7% showed the flush terminal plane, followed by the distal step in 21.2% and mesial step in 26.1% of cases. There were no significant differences in genders. The most prevalent relationship between deciduous second molars was the flush terminal plane, with equal distribution in all age groups. The frequency of distal step decreased with age, more frequently recorded in the 3 to 4 year age group. The mesial step was mostly recorded in 3-4 and 5-6 year groups.

**Conclusion.** The flush terminal plane is the most frequent molar relationship, followed by mesial and distal step. Prevalence of distal mesial step significantly decreased with age, while mesial step relationship showed the tendency to increase. The flush terminal plane showed little change with age.

## Introduction

 The normal primary dentition can serve as the predictor of developing normal permanent occlusion. The eruption of primary second molars completes the formation of primary dentition and determines the future position of permanent first molars.^1^ Therefore, early diagnosis and treatment could prevent maxillofacial disorders and dysfunction in adulthood.^2^ With preventive and interceptive measures we can change the abnormal growth pattern of the face, thus improve psychological and social well-being, which can be altered by different types of skeletal and dental disturbances.

 Malocclusion is the disturbance of relation between teeth of the same or opposing dental arches and is one of the most frequent disorders among children after dental caries.^3^ However, the main goal for the pediatric dentist is to identify normal and abnormal situations during primary dentition, plan and assess preventive and interceptive measures.

 Foster and Hamilton evaluated a wide variety of occlusal conditions in primary dentition and stated normal occlusion as having the following characteristics: spaces between anterior teeth, primate spaces, no or minimal overjet and overbite, distal surfaces of second deciduous molars on the same plane (flush plane), ovoid arch form.^4,5^Primary molar relationship was studied by Baume and is classified in three main types: flush terminal plane, distal and mesial steps.^6^ The terminal plane is determinant for developing occlusion, as the posterior surface of the distal root of the primary second molar directs the eruption path for the first permanent molar.^3^

 As the primary dentition transits to mixed dentition, the flush terminal plane can develop to end-to-end or Class I permanent molar relationship with the forward growth of the mandible. Distal step mostly develops to Class II permanent molar relationship, while mesial step can lead to Class I or Class III permanent molar relationship. According to the studies, the prevalence of mesial step increases by the age of 6 years age, providing more favorable contacts between erupting permanent first molars, while the frequency of flush terminal plane reduces by time. However, Profit has reported, that children with mesial step during primary dentition, are more predisposed to develop Class III malocclusion. According to Dutra et al, 56% and 44% cases of the flush terminal plane will develop into Class I and Class II occlusion in permanent dentition. Mesial step in most cases (76%) will develop in Class I, in the least cases Class III. The distal step does not self-correct and develops onto Class II occlusion, thus early evaluation and interceptive treatment is necessary.^7^

 The articles and surveys conducted on primary or deciduous dentition have generally been in descriptive format, serving to describe such things as normal occlusion and molar relationship assessment. The range of the prevalence varies between 21.0% to 88.1%.^8-13^ There is a lack of such surveys in Georgia.^14,15^ Obtaining data about occlusal status could help adopt oral health projects and prevent the development of dental deformities associated with early childhood malocclusions.^16,17^ Therefore, the aim of this study was to collect data about the prevalence of malocclusions based on the terminal planes among the preschool children of Tbilisi, Georgia. Retrieved data will help us to raise awareness, plan preventive measures and write recommendations for pediatric and general dentists, pediatricians, and other health care workers.

## Methods

 The capital of Georgia, Tbilisi was selected for study location. The survey was conducted from March 2019 to June 2019. Multi-stage cluster sampling with preliminary stratification was applied to get a sample of preschoolers. In each stratum, kindergartens were selected using the PPS (probability proportional to size) method.

 A total of 396 children aged 3-6 years, with an equal number of male and female subjects, were examined in kindergartens drawn from ten districts of Tbilisi. Children were examined on-site. Occlusion was checked in maximum intercuspation, or if necessary mandible was manipulated into centric relation. Data inclusion criteria included the existence of fully erupted primary dentition, no partially or fully erupted permanent teeth, and without a history of any orthodontic intervention. Criteria for exclusion were the presence of any permanent teeth, loss of primary teeth, extensive tooth material damage that could affect the mesiodistal or occlusogingival dimension of a tooth, therefore, affect the occlusal characteristics, tooth agenesis, congenital disorders causing maxillofacial deformities, or severe illnesses, children who couldn’t cooperate with the researcher.

 Written permission from the kindergarten governing agency and participating children’s parent’s or legitimate guardian’s written informed consent was obtained in each case. The study was approved by the Tbilisi State Medical University Biomedical Research Ethics Committee (re: 2015-0012 N1-2018/66. 17.04.2018).

 The study was held in the classroom provided by school authorities. Standardization was applied to the data collection technique and method, under the supervision of a senior operator (T.M). The examination was performed by one examiner (E.G), who investigated the relationship of primary second molars, presence of flush, mesial or distal step under natural light in the presence of their parents/guardians, using disposable gloves, mirror and scapula.

 Baume’s classification was used to evaluate the primary second molar’s distal surface relationship:

1. Flush terminal plane (Class I or neutral occlusion) - when the distal surfaces of upper and lower second molars are in the vertical plane.
2. Distal step (Class II) - when the distal surface of the lower second molar is distal to the upper in centric occlusion.
3. Mesial step (Class III) - when the distal surface of the lower second molar is mesial to the upper in centric occlusion.
4. Asymmetric molar relationship

The molar relationship was recorded on right and left sides of dental arches, in the case of asymmetrical relationship, decision and record were done in favor of flush terminal plane.

 The obtained data were processed and analyzed using the SPSS v21.00 (Statistical Package for Social Sciences). The significance level is 0.05 for all statistical tests. Independent - samples T-test was used to compare the variables.

## Results

**Figure 1:**
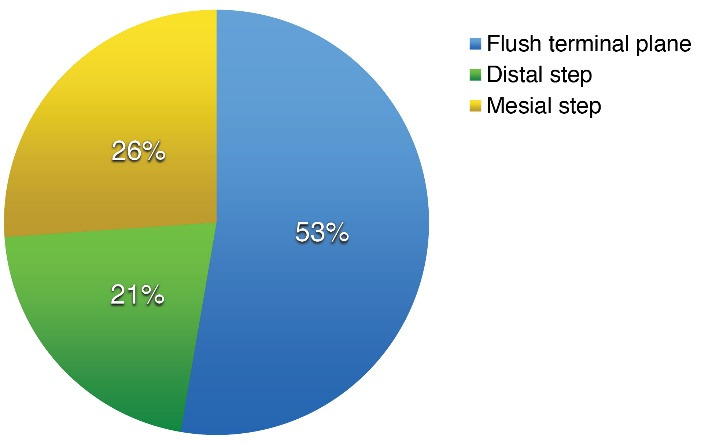
Distribution of primary molar terminal plane relationship.

 A total of 396 children aged 3 to 6 years, with an equal number of male and female subjects, were examined. Out of the population, 52.7% showed the flush terminal plane, followed by a distal step in 21.2% and a mesial step in 26.1% of cases. There were no significant differences in genders (Figure 1).

 The overall prevalence and age-wise distribution of various occlusal molar relationships in the primary dentition of children aged 4 to 6 years are shown in Table 1.

**Table 1:** Age-wise prevalence of occlusal parameters in the primary dentition in children in three age groups.

**Age groups** **Terminal plane relationship**	**3-4 years old n(%)**	**4-5 years old n(%)**	**5-6 years old n(%)**	**Total n(%) **
Distal step	5160 (39.9)	4218 (32.6)	3559 (27.5)	12937 (100.0)
Mesial step	3348 (24.5)	4631 (33.8)	5690 (41.6)	13669 (100.0)
Flush plane	11174 (34.4)	10833 (33.4)	10433 (32.2)	32441 (100.0)
Total	19682	19682	19682	59047

^*^P<0.05 was considered significant

 As seen on the table, the flush terminal plane was more prevalent in all age groups with equal distribution. The frequency of the distal step decreased with age, more frequently recorded in the 3-4 year age group. The mesial step was mostly recorded in 3-4 and 5-6 year groups. The revealed differences in age groups were found to be statistically highly significant.

## Discussion

 The primary dentition is the predictor of future permanent dentition. It is fully established by the age of 3 years and lasts till 6 years when it starts the complicated process of transition to permanent dentition. Due to the transition of sagittal occlusal discrepancies to permanent dentition, there is a need of raising awareness of the role of primary dentition. The lack of epidemiological data in Georgia has determined the necessity of such research. Our study aimed to evaluate the primary molar relationship in different age groups among Georgian preschoolers. Our results revealed that the majority of children had a flush terminal plane (52.7%), followed by mesial (26.1%) and distal step (21.2%).

 The literature overview shows that the epidemiological data regarding primary molar relationship is different among different countries and ethnicities. These differences can be due to different methodology, racial specifications, eating habits, and other characteristics. According to various studies, the most frequent molar relationship is the flush terminal plane, followed by mesial and distal step, which is in line with our study.^18-20^

 A study by Yilmaz et al showed the highest rate (88.29%) of the flush terminal relationship rate among 3- to 6-year-old Turkish children.^21^

 According to Anu et al and Kumar et al, the most frequent relationship among African, American, and European preschoolers was the mesial step, the same findings were also mentioned in studies of Anu et al, Kumar et al.^21,24^

 In our study mesial step was revealed 26.1% of cases, while in other studies like Fernandes and Bhayya et al, the prevalence was 43.3% and more.^22,24^

 Other studies showed a much more low distribution of distal molar relationship, as the study by Vegesna et al.^18^

 Based on our findings, it is evident that the prevalence of distal mesial step significantly decreased with age, while the mesial step relationship showed the tendency to increase from the smaller age group to bigger. At the same time, the prevalence of flush terminal plane showed little change. This tendency was supported with some other studies, while some of them showed no changes in molar relationship throughout primary dentition.^22^ Changes in the molar relationship may be connected with the eruption of permanent first molars and sagittal growth of the mandible.^24^

 Contrary to our findings, Nanda et al have stated, that the distal step molar relationship would not change during primary dentition and always transferred to the same type of malocclusion in the permanent dentition.^25^ Longitudinal study done by Ravn made this hypothesis more reliable.^26^

 The changes happening during the transition process of primary to permanent dentition were evaluated by Bishara et al.^1^ According to his observations, in 54% of cases flush terminal plane developed into Class I, 44% of cases into Class II. The distal step always developed into Class II, without the chance to self-correct, therefore early orthodontic treatment should be advised. The mesial step would mostly transit to Class I, rather than Class II. Whether the mesial step would transfer to Class III permanent molar relationships, would be much more dependent on the magnitude of the step.^1^ Other longitudinal studies such as done by Onyeaso and Isiekwe have reported the development of Class I from most of the cases with the flush terminal pane.^27^

 As stated by Johannsdottir et al mesial step was further connected with Class I (91%), Class II (1%), and Class III (8%).^28^ Nanda et al thought that the mesial step was the most favorable anteroposterior relation between molars, with the highest rate of developing Class I.^25^

 The distal step mostly developed to Class II molar relationship with the increasing rate throughout the primary dentition period.^19^ It can be stated that the distal molar relationship is the only stable occlusion that is maintained during the primary dentition period and is always transferred to permanent dentition.

 In our study, we stated a high frequency of flush terminal and mesial step relationships and we may predict the future high prevalence of Class I. Our study did not show differences between the genders.

 Epidemiological studies in primary dentition are important to understand and predict future development of permanent dentition, also to evaluate orthodontic intervention dates in this particular age group. All conditions should be registered and parents given appropriate information about future treatment options.

 Early diagnosis, preventive measures, and treatment might prevent further development of maxillofacial anomalies, deformities, and functional disorders. Additionally, we can manage facial growth at an early age to avoid physiological disturbances associated with malocclusion and maxillofacial anomalies.

## Conclusion

 After the evaluation of 396 Georgian preschoolers, the following conclusions can be drawn:

- The flush terminal plane is the most frequent molar relationship, followed by mesial and distal step.
- Prevalence of the distal step significantly decreased with age, while the mesial step relationship showed the tendency to increase. The flush terminal plane showed little change with age.
- Malocclusion among Tbilisi preschoolers is not gender specific.
- The present study was a limited cross-sectional study that needs further longitudinal research in this field.

## Acknowledgments

 The authors wish to acknowledge the help provided by the head of Ivane Javakhishvili Tbilisi State University dental health department: D.M.D., Ph.D., D.M.Sci Vladimer Margvelashvili. We would also like to thank the staff of the Tbilisi Kindergarten Agency for cooperation.

## Authors’ Contribution

 TM and MK and EG conceived and designed the work, collected the data, contributed to data analysis, and wrote the paper. All the authors contributed to the critical revision of the manuscript and have read and approved the final version to be published.

## Funding

 Self-funded.

## Ethics approval

 The study protocol was approved by the institutional ethical committee of Tbilisi State University, Georgia.

## Competing interests

 The authors declare no conflict of interests related to the publication of this work.
